# Pre-Natal Education

**Published:** 1886-02

**Authors:** I. B. Comings


### PRE-NATAL EDUCATION.

I. B. COMINGS, M. D.

It is sometimes stated in the biographies of some men or women, that the person described could not help being what she or he was. This remark arises from the fact that the ancestery of the person described, was so pure and well organized, that we could expect nothing else than almost perfection, or the reverse of this, if the ancestry were vicious and uncultivated.

The trite remark, "blood will tell," is a universal law. In the animal it is noticed, and often traced through generations. In the physical form and constitution it is so conspicous and so desirable to perpetuate; that the most lavish expense will often be incurred to accomplish it. But mental qualifications and characteristics are just as truly transmitted as the physical, for the mental has to work in and through the physical organization, hence in the vital organization we are to look for all the phenomena of heredity.

Going a little back of even pre-natal conditions, we know that the future of our offspring is largely dependent on the mental and moral status, as well as the physical condition of the parents at the time of the creative impulse.

For we see that the child often favors the father more than the mother, and seems to posses the traits and the constitution of the male, far greater than the mothers; this shows that the vital and mental characteristics of the male is impressed on the offspring by some psychic force rather than physical, for it is said by our best physiologists that 999-1000th of the constituent of the organization of the foetus is derived from the mother, it must follow then that if this influence of the male parent is not made upon the mind of the mother, little will resemble the father. But more than this, we were taught a few weeks ago by Mr. Dawborn in that most admirable lecture on the "Unborn Man," that even impressions and character may be stamped on the fœtus by the mother from a previous husband, as was proved by those well-known instances of the Quaggy and the offspring of the mother with the colored husband proving conclusively that there is an impressive power above and beyond the physical organization, and that it must be spiritual or psychic in its nature.

Again it has been shown that the mental antagonism of a meternal parent to the will of the father, and the bitter feelings often engendered between mismated parties, will often develop a corresponding impulse in the child so that it cannot help being what it is, for its fate was really determined before it was born. How much sympathy and forbearance this fact ought to have on our minds, in the treatment of the unfortunate sons of men who are suffering in our prisons and public institutions. We ought to change our whole penal judiciary; instead of prisons and corporal punishments, we ought to have hospitals of reform, and systems of education to counteract these natural tendencies, and cure these evils rather than punished them.

We all acknowledge the duty of parents to attend to the proper education of the child from its birth till it is grown up, and to attend to its development that its body and mind may be properly educated; but how little is thought of its pre-natal education, or how little is known or even conceived of its formation and growth while the very soul of this immortal being is building up the structure which is to contain this living principle, and which is to mould and shape its almost infinite powers, for good or evil, for all future time and even for eternity.

From the earliest periods of physiological investigation it has been known that the state of the mother's mind has more or less to do with the embryo.

Dr. Carpenter, our best authority on physiology, says: "during the entire period of gestation, the new being is deriving its nutriment exclusively from the blood of the mother, and that the condition of this fluid, in relation to her own processes of nutrition and secretion, are subject to a very marked influence from her own mental status; it cannot fairly be thonght improbable that the developmental processes of the embryo should be powerfully effected by strong emotional excitement on her part." And further on he says, "the influence of both parents, on the constitution of their offspring is strikingly manifested, not merely in the admixture of their characters, but there are diseases very prone to appear in successive generations, such as scrofula, gout, syphillis and insanity, and in no case is this more obvious than in the influence of alcoholic excesses on the part of one or both parents in producing idiocy and pre-disposition to insanity."

Such are the teachings of physiology. We give some instances which have came under our own obsevations, which should serve to impress on mothers the importance of beginning the education of their offspring even before their birth.

We are acquainted with a family of ten children, and intimate with the parents; they were both healthy. The marriage was one of pure affection, and the utmost congeniality existed between them. During the first pregnancy of this mother she was busily engaged in sewing and learning to cut and make up clothing; her whole mind was fully occupied during that period—as she had very little experience in these matters before marriage; for she suddenly became a wife from the school girl.

The first child, a daughter, very early manifested an aptitude and skill in cutting and making doll dresses, was an expert with the needle far above ordinary cases, and this seemed intuitive and almost without instruction. The second child of this mother was born with a "*spina bifida*," and lived but a few days. This bification of the spine was supposed to be the result of stepping upon a kitten, and breaking its back, for it gave the mother a severe shock and such an impression on the mind as to produce that result. The third pregnancy was not marked by any peculiar condition of the parent and the son born manifested no peculiar traits or characteristics that were particularly noticable. The fourth was a boy with a religious turn of mind, was early in the church, and had many peculiarities which can be readily traced to the state of mind of the mother while she carried him. The fifth child was a daughter who from her earliest childhood manifested a peculiar fondness of housekeeping, for fixing up and putting in order the furniture, etc. This can be attributed to the occupation of the mother; for some months previous to her birth, she was engaged in settling in a new house, and her mind was fully occupied in such labors as moving into a new house, arranging new furniture, etc., would require. The sixth child, only lived one year, but sufficiently long to show a good musical ear, and a remarkable fondness for singing and piano music. He would move his little hand along the keys to vary the sound, quite different from most children of that age. Now the mother's time, previous to this birth, was occupied with music teaching and frequently playing.

These parents having noticed the various traits in their children, as we have described them, thought they would test the truth of the theory they had formed, so when the seventh pregnacy was known, the mother kept herself in a most equable and pleasant frame of mind, free from excitement of any kind. She tried to be happy and joyful during the whole period, visited places of amusement more than usual, and was very observant of everything that could contribute to her happiess. As the result of this course when the daughter came along, she was a large, healthy, beautiful child, and as she grew up to women's estate, she manifested the highest graces of mind, and the perfect womanly qualities, a disposition hardly to be improved, and a loving nature that is almost the admiration of all; fully showing the happy influence of the mother's mind upon the constitution and temperament.

The eigth child possesses the peculiar, happy and loving trait of the mother, but as she was not peculiary exercised, nothing is particularly noticeable. The ninth child was born after the mother had been sick with chills and was in poor health, so this daughter was of a more fragile constitution, seemed to possess much of the mother's weakness manifested during this period.

The tenth child is yet young and has not manifested any peculiarity; she may not, as there was nothing observable in the condition of the mother. In another family of seven sons, there was one very erratic and uneasy and dissatisfied with every thing; so different from the other members of the family that it astonished every one.

In conversation with the mother of those sons, she informed me that during the pregnancy with this son, she was exceedingly troubled, or her husband was, by pecuniary losses. She was harrassed in various ways and much afflicted, so that she thus accounted for the peculiarities of this child.

Another case which came under our observation, was that of a pious orthodox mother, who was greatly exercised on religious matters and attending protracted meetings during the whole period she was "*enciente*," and this child at the four years of age, was remarkable for great gift of prayer and religious conversation, and astonished every one for his preaching talent. He died at the age of six years, ripe for heaven, as every body said. One other instance of these pre-natal tendencies and we leave this part of our subject for deeper hereditary pre-dispositions. We called once to see an infant but a few months old, which seemed to manifest some most singular symptoms. We were at a loss to account for them. We found our prescriptions of little profit; as this child grew up, it had all the symptoms and actions of a drunkard. In all his movements there was a perfect imitation of the drunken father who subsequently became a most exemplary Chistian and tetotaler. He said to me once while this son was passing along the street near by, "There you see a judgement on me for my intemperance." Will not a knowledge of this subject and a suitable *practicable observance* of such rules and regulations as will secure the attainment of its advantages be of incalculable value to the human race? Does it become us to give so much attention to the breeding of horses and cattle and yet neglect that of the human race? If the *physical* development of the body is of great moment, how much greater is that of the *mental* and *moral?* And when we remember how difficult it is to change or reform those who are *born* with bad or vicious tendencies it must be evident to all that this early education of the *embryo* is of far more importance than any subsequent training.

Having thus shown what the influence of pre-natal agencies may have upon the physical organization, we will look a little deeper into the mental and physical influences which are at work at all times.

Physiology teaches us that constitutional diseases are not only transmitted through the *physical* constitution, but that this heredity, so called, is exerted upon the *spirit* entity or *soul* of the embryo. We are taught that the whole human body is changed every seven years, that every particle that existed seven years ago has disappeared from the human organization, and that the whole body is a "new creation." Prof. Huxley says, "so constant and universal is the absorption, waste and reproduction, that it may be said with perfect certainly that there is left in no one of our bodies at the present moment a millionth part of the matter of which they were originally formed. *Bone* once formed does not remain during life, but is constantly dissappearing and being replaced in all its parts." If bone, than the softest tissues. Now if this is so, how is it possible to transmit diseases in the blood? And yet we know that disease is transmitted. There must be a deeper heredity, or an impression made on something that is unchangeable; it must be the soul-entity that becomes effected, that principle or vital germ on which all humanity is founded.

If we will remember that it is a scientific proposition, that the *mental* powers are the *real* entities, and not subject to change, like the materials of the physical organization ; that it is through these mental an physical characteristics, that diseases or peculiarities are transmitted from generation to generation; this is evident from an accepted fact in physiology (before referred to) that nine hundred and ninety-nine one thousandths of the infant's physical body is derived from the maternal source, and yet you see the offspring resemble both father and mother alike ; indeed if there is any difference it is in favor of the father. You see then, it would not be logical to infer that this hereditary taint came from the mother's organization, for if it was so, the child would resemble the mother almost exclusively.

It is this mental or physical impress which explains "the mother's mark" and pre-natal peculiarities which we have mentioned above.

There must be a mental electricity through which these changes are effected, it must permeate the whole organization of the mother, like the subtle currents through the electric battery, and what impresses this energy on the mother affects the fruit of the womb. This must be the way that the impressions are made, for there are no *nerves* connecting the mother with the fœtus.

Dr. Carpencer says; "Influences and shocks cannot impress the fœtus through nerve media because there is no system of nerves connecting the mother and fœtus, and physiologists are in doubt of the possibility that impressions sufficient to produce malformation, etc., can be accomplished through the circulation of the blood. So we are forced to look for another source of this action, and it must be that psychic impression which is made on every vital germ cell or ovule. For this life pattern, not being liable to metamorphosis or mutation like the blood, tissue and bone, is the only basis on which the transmission of character can find conduction from generation to generation.

This spirit being, or vital mental organization, is all there is about a human being or animal which is not constantly undergoing mutation or substitution from the first pulsation to the last.—*Olive Branch.*

